# Effects of Sodium Tripolyphosphate Addition on the Dispersion and Hydration of Pure Calcium Aluminate Cement

**DOI:** 10.3390/ma16083141

**Published:** 2023-04-16

**Authors:** Benjun Cheng, Hao Feng, Erbolat Armia, Hongli Guo, Shaowei Zhang, Haijun Zhang

**Affiliations:** 1 School of Energy Science and Engineering, Central South University, Changsha 410083, China; 2 Sinosteel Group Luoyang Refractories Research Institute Co., Ltd., Luoyang 471039, China; 3 College of Engineering, Mathematics and Physical Science, University of Exeter, Exeter EX4 4QF, UK; 4 The State Key Laboratory of Refractory and Metallurgy, Wuhan University of Science and Technology, Wuhan 430081, China

**Keywords:** sodium tripolyphosphate, pure calcium aluminate cement, hydration behaviour, dispersion, potential principle

## Abstract

In this paper, the effect of a sodium tripolyphosphate (STPP) addition on the dispersion and hydration of pure calcium aluminate cement (PCAC) was investigated, and the corresponding mechanism of effect was studied. The effects of STPP on the dispersion, rheology, and hydration processes of PCAC and its adsorption capacity on the surface of cement particles were analysed by measuring the 𝜁-potential on the surface of cement particles, the changes in the concentrations of elemental P and Ca^2+^ ions in a solution at different STPP additions. The experimental results show that STPP easily complexes with Ca^2+^ ions to produce the complex [CaP_3_O_10_]^3−^ adsorbed on the surface of cement particles, which changes the potential on the surface of cement particles and increases the electrostatic repulsive force between cement particles, thus improving the dispersion and rheology of cement. At the same time, the contact area between cement particles and water is reduced, which hinders the hydration process and makes the time of hydration process longer. A comprehensive analysis shows that the best effect of STPP on pure calcium aluminate cements is achieved when the addition of STPP is 0.2%. This study can provide a reference for the addition of water-reducing agents in refractory castables as well as improving the quality of refractory materials.

## 1. Introduction

Calcium aluminate cements (CAC) are often used as binders for refractory materials due to their excellent properties, such as shock resistance and high temperature resistance [1,2]. When pure calcium aluminate cements are used for refractory castables, a certain amount of water-reducing agent is usually added, which has a dispersing and lubricating effect on the cement particles and improves the compatibility of the cement slurry. In addition to this, it reduces the amount of water used, improves the fluidity of the slurry, and increases the strength of the cement slurry hardening body. The rheological properties, dispersion, and hydration processes are important properties of cement, which affect the working time, efficiency, and performance of the castables. The chemical phase composition of the cement [3,4,5], the water-reducing agent added to the castables, and the construction temperature [6,7,8] are all factors that affect the hydration process. In refractory castables with aluminate cement as a binder, high-efficiency water-reducing agents, such as sodium tripolyphosphate (STPP) and sodium hexametaphosphate (SMHP) are usually used, and in refractory castables with pure calcium aluminate cement (PCAC) as a binder, a STPP high-efficiency water-reducing agent is usually used. STPP is a chain-concentrated phosphate that is readily soluble in water, has a significant chelating ability for metal ions, and has a strong synergistic effect with surfactants [9]. Due to its phosphate group [10,11,12], STPP is active towards cationic groups, including amino and metal ions. These excellent-use properties can improve the construction properties and enhance the mechanical strength of the castables.

In recent years, researchers have carried out a series of studies on the role of water-reducing agents in cements. Ma and Tan [13] studied the mechanism of action of different water-reducing agents on cement hydration and found that water-reducing agents can inhibit cement hydration and hinder the precipitation of mineral primary phases, thus slowing down the hydration process. Shang and Hou et al. [14] studied the hydration behaviour of calcium aluminate cements and obtained the hydration of calcium aluminate cements at different temperatures. Wang et al. [15] found that STPP selectively reacted with Ca^2+^ ions to form stable complexes, reducing the adverse effects of metal ions (e.g., Ca^2+^) on the dispersion of diaspore and kaolinite. Papo et al. [16] investigated the dispersion properties of STPP and found that STPP exhibited shear dilution behaviour at all concentrations over the entire range of shear rates studied. Several researchers have tested the ability of STPP to chelate Ca^2+^ ions, proposed various test routes, and analysed the factors affecting the chelating ability [17,18,19,20,21]. Yang Ping [22] studied the adsorption behaviour of STPP on the surface of silicate cement particles and the mechanism of action of the retarder and found that sodium tripolyphosphate can form complexes with Ca^2+^ present on the surface of cement particles, altering the surface electrical properties of cement particles, while inhibiting Ca^2+^ dissolution, reducing the concentration of Ca^2+^ in the liquid phase, and delaying the exothermic hydration as well as the formation of hydration products.

Although many studies have been carried out on STPP and PCAC, there are no studies on the effects of STPP on the dispersion and hydration of CAC, and there is a lack of systematic studies on the mechanism of STPP retarding and a vague understanding of the intrinsic link between the adsorption behaviour, rheology, hydration, and retarding of PCAC. These elements are all unresolved problems in the series of water-reducing agent and cement action. To address these issues, this study on the action of STPP on PCAC was carried out, which can provide a reference for the selection of a suitable water-reducing agent for refractory castables and will also contribute to an in-depth understanding of the effect of STPP on the dispersion and water-reducing properties of PCAC-bonded castable systems. In the course of this study, the ζ-potential corresponding to the mixture of different contents of STPP with pure calcium aluminate cement, the P-element and Ca^2+^ concentrations in the filtrate, and the shear rate of the cement slurry were measured experimentally. Based on the experimental data, the dispersion, adsorption, and rheological properties of PCAC in the presence of STPP can be analysed. The hydration behaviour of PCAC was analysed with the help of X-ray diffraction and scanning electron microscopy (SEM), and the mechanism was discussed by reviewing relevant data. After processing the experimental data, the mechanism of the effect of STPP on the use properties of PCAC was finally concluded, and the optimum amount of STPP to be added to PCAC refractory castables at room temperature in the summer was also analysed.

## 2. Experimental Section

### 2.1. Raw Materials

All the raw materials required for this study are pure calcium aluminate cement (PCAC) and sodium tripolyphosphate (STPP). PCAC was supplied by the Sinosteel Group Luoyang Refractories Research Institute Co., Ltd. (Luoyang, China), and STPP was supplied by from Luoyang Hexin Refractories Co., Ltd. (Luoyang, China). The chemical composition of PCAC is detailed in Table 1.

### 2.2. Preparation of Experimental Samples and Test Methods

#### 2.2.1. Determination of STPP Adsorption

A total of 10 g PCAC was weighed and mixed with 200 mL of STPP solution at 0%, 0.05%, 0.1%, 0.2%, and 0.4% concentrations and then stirred with a glass rod for 10 min to mix each solution well. The concentration of P and Ca^2+^ in the filtrate was measured using plasma absorption spectroscopy (ICAP 6000 SERIES, Cambridge, UK) to estimate the amount of STPP remaining in the filtrate and to calculate the amount of STPP adsorbed on the surface of the cement particles [22].

#### 2.2.2. Determination of the Rheological Properties of PCAC Slurries

PCAC was homogeneously mixed with water at a ratio of 7/3 by weight; then, 0%, 0.05%, 0.1%, 0.2%, and 0.4% of STPP were added to the mixture, and the rheological behaviour of each slurry at 37 °C (summer room temperature) was examined using a coaxial cylindrical-shaped (ST22-4V-40 system) rotational rheometer (MCR301, Anton Paar, Styria, Austria). During this test, the shear rates ranged from 0.1 s^−1^ to 1000 s^−1^.

#### 2.2.3. Determination of STPP Adsorption

An appropriate amount of 10 g of PCAC was weighed and mixed with 200 mL of STPP solution at 0%, 0.05%, 0.1%, 0.2% and 0.4% concentrations, and stirred with a magnetic stirrer for 5, 10, 15 and 20 min. The diluted suspension of 10 mL was removed and the potential values on the surface of the cement particles were measured with a ζ- potentiometer (ZetaProbe, LWL Development Limited, Hong Kong, China) [23]. During the experiment, the pH value of the system was also measured to be within the standard range, proving that the experiment was reliable.

#### 2.2.4. Analysis of PCAC Hydration Behaviour Using XRD and SEM

Amounts of 0%, 0.05%, 0.1%, 0.2% and 0.4% of STPP were mixed with 2 kg of PCAC, and the appropriate amount of water was added during the mixing process. The mixture was then poured into a 40 mm × 40 mm × 160 mm mould, shaped by vibration and left to stand for 24 h at a constant temperature of 37 °C (simulating summer room temperature), and demoulded to obtain the experimental samples. All samples were oven-dried at 100 °C for 24 h. Then, the samples were smashed into 200 mesh powder for the measurement. The samples were analysed for mineralogical information by X-ray diffraction (XRD) (PANalytical, Empyrean, The Netherlands). Measurements were performed under vacuum and at room temperature. The scan-angle ranges of the instrument are from 10° to 70° with a step size of 0.03, counting time of 1.5 s per step, power of 3 kW, high voltage stability of 0.005%, and goniometer accuracy of 0.0001°. The sample microstructure was observed using a scanning electron microscope (SEM) (Nova400NanoSEM, Amsterdam, The Netherlands) with the instrument operating at 20 kV with a resolution of 8 nm at 20 kV high voltage and an energy spectrometer resolution of 133 eV. The electron beam was focused on the sample surface with a magnification of 3000 times, and the sample surface images were recorded in this case. Finally, the original diffraction data were analysed, and the mineral content was determined using jade software; the mineralogical information from XRD was used as a reference for the identification of the minerals contained in the samples with the help of SEM.

## 3. Results and Discussion

### 3.1. Adsorption Behaviour of STPP

**Figure 1:**
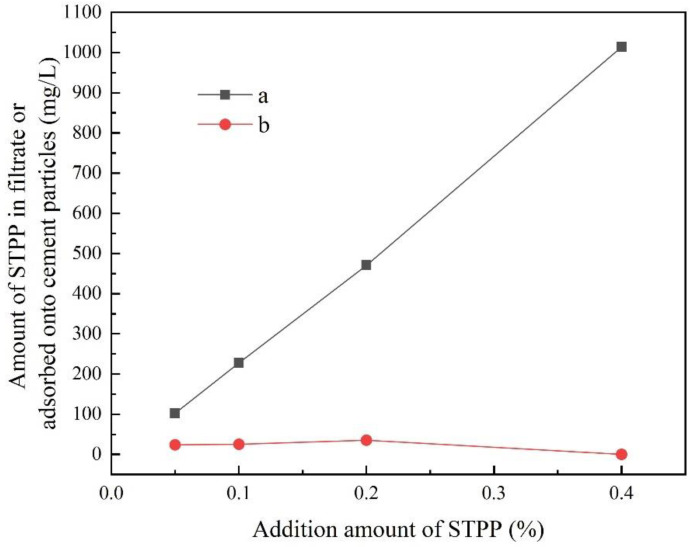
Amount of STPP in the filtrate after 10 min of stirring (a) and adsorbed onto cement particles after 10 min of stirring (b).

**Figure 2:**
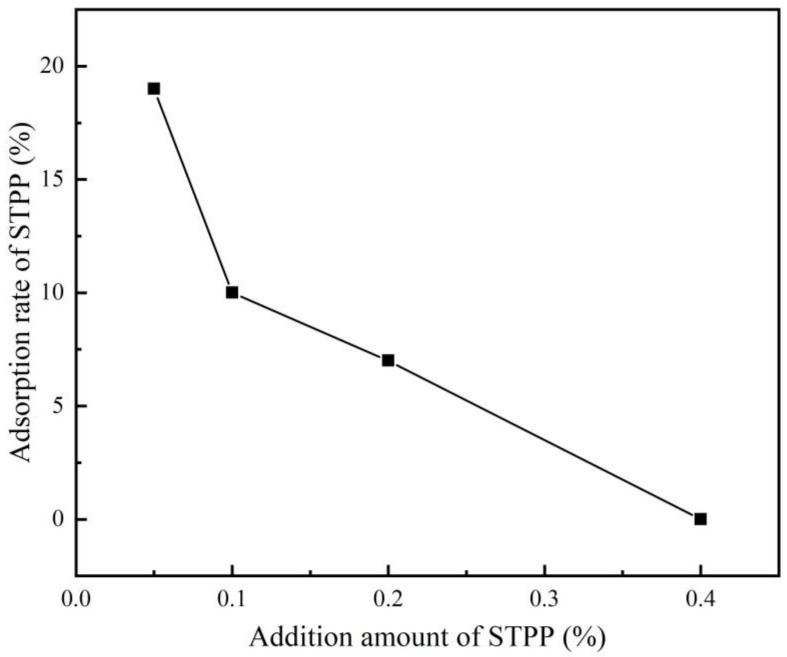
Adsorption rate of STPP after 10 min of stirring, as a function of the addition amount of STPP.

Figure 1 shows the variation of the P-element concentration in the solution with the amount of STPP added after 10 min of stirring. The concentration of elemental P in the filtrate was measured using ICP, and the amount of STPP adsorbed on the cement particles could be estimated based on the P concentration remaining in the filtrate. According to Figure 1, it can be seen that the amount of STPP adsorbed on the surface of the PCAC particles is low. Figure 2 further illustrates the variation of the STPP adsorption rate (i.e., the ratio of the amount of STPP adsorbed onto the cement particles to the total amount of STPP added) with the amount of STPP added. When the STPP addition was 0.05%, the adsorption rate of STPP was 19%. However, it decreased to 0% when the amount of STPP addition was increased to 0.4%. The decrease in the amount of STPP in the solution may exist in two situations: (1) STPP chelated with Ca^2+^ to form a complex, which adsorbed on the surface of cement particles and formed an electric layer structure, and (2) a phosphate that is insoluble in water was generated. According to the study of Ma Baoguo et al. and Rongjia, it is known that the reaction between STPP and PCAC is consistent with the first case, and the degree of reaction is different at different concentrations. When the concentration is small, it is very easy to generate complexes, which are adsorbed on the surface of cement particles, and reduce the amount of STPP in a solution; when the concentration is large, the generation of complexes is inhibited and the amount of STPP in solution changes less.

**Figure 3:**
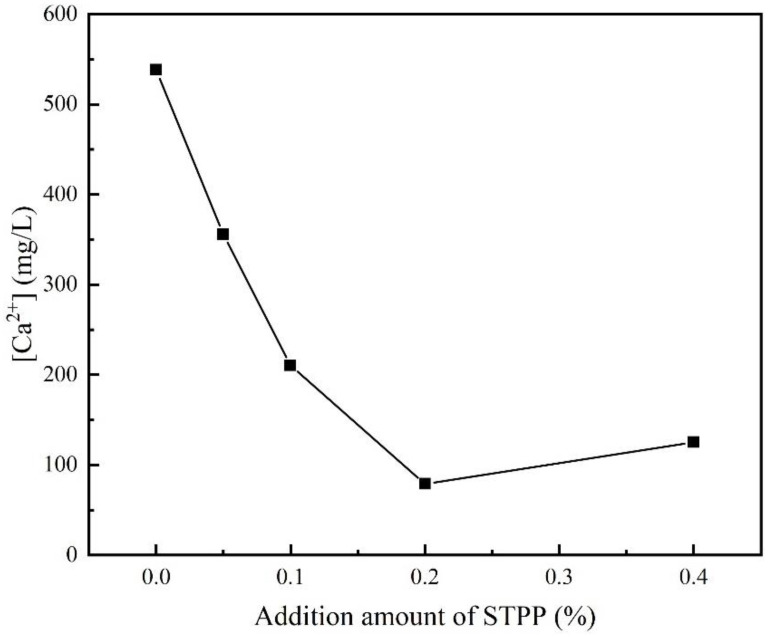
Effect of STPP addition on Ca^2+^ concentration in the filtrate after 10 min of stirring.

Figure 3 shows the variation of Ca^2+^ concentration in the filtrate with the amount of STPP added after 10 min of stirring. As can be seen from Figure 3, the Ca^2+^ concentration was 538 mg/mL without the STPP addition, but when the amount of the STPP addition was increased to 0.2%, the Ca^2+^ concentration decreased to 79.14 mg/mL. When the STPP addition was 0.4%, the calcium ion concentration increased to 125.16 mg/mL. As shown in Reaction (1), the STPP can react with Ca^2+^ to form [CaP_3_O_10_]^3−^ ions. The above results indicate that the degree of chelation of STPP with Ca^2+^ varies at different concentrations. At lower concentrations, the addition of STPP inhibited the dissolution of Ca^2+^, and the degree of inhibition decreased at higher concentrations. When the STPP dosage was increased in the range of 0–0.2%, the Ca^2+^ concentration in the solution started to decrease, which meant that STPP reacted with Ca^2+^ chelation to form the soluble complex Na[Na_2_Ca(P_3_O_10_)] [24,25], and the water-soluble [CaP_3_O_10_]^3−^ ions in the complex were adsorbed to the surface of the cement particles and then precipitated with the cement. When the dose of STPP was further increased to 0.2–0.4%, the residual Ca^2+^ concentration started to increase, which, according to Zhou [15,18], was related to the saturation of the electro-layer ion concentration, at which time the [CaP_3_O_10_]^3−^ ions decreased and the amount of STPP adsorbed to the cement particles decreased accordingly. The best water-reducing effect was achieved at the STPP addition of 0.2%.

[P_3_O_10_]^5−^ + Ca^2+^ →[CaP_3_O_10_]^3−^ (1)

### 3.2. ζ-Potential of Cement Particles

**Figure 4:**
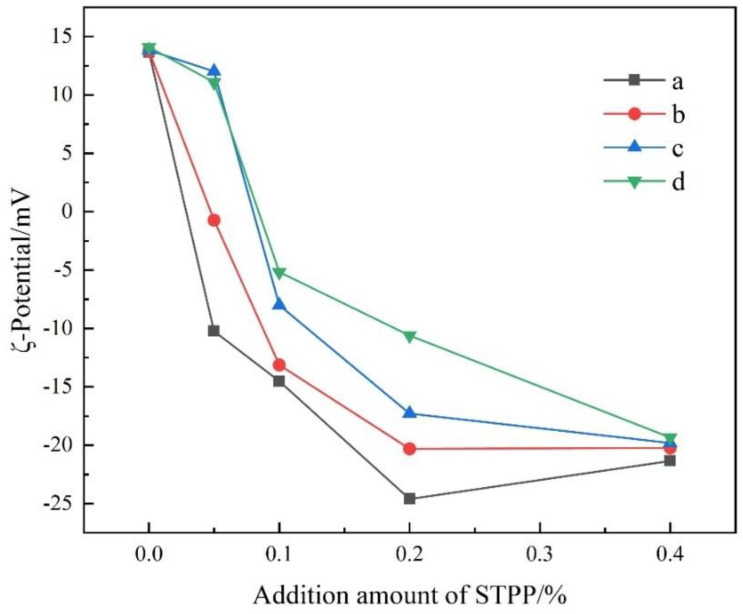
ζ−potential of cement particles as a function of the additional amount of STPP and stirring time: (a) 5 min, (b) 10 min, (c) 15 min, and (d) 20 min.

Figure 4 shows the variation of ζ-potential of cement particles with an additional amount of STPP and stirring time. After stirring for 5 min and 10 min, the ζ-potential on the surface of the cement particles changed from positive to negative and decreased gradually when the additional amount of STPP was 0–0.2%; when the additional amount was 0.2–0.4%, the ζ-potential increased to −21.35 mV and −20.23 mV, respectively, and reached a minimum of −24.01 mV and −20.30 mV at the additional amount of 0.2%. After stirring for 15 and 20 min, the ζ-potential on the surface of the cement particles changed from positive to negative and decreased gradually with the increased addition of STPP. With the same amount of STPP added, the shorter the stirring time, the stronger the ζ-negative potential intensity. The variation pattern of ζ-potential after 10 min of stirring is consistent with the variation pattern of Ca^2+^ concentration in the filtrate in Section 3.2.

The abovementioned variation pattern has the following explanation. When STPP is added, water-soluble [CaP_3_O_10_]^3−^ is formed due to the chelation of STPP with Ca^2+^. The Ca^2+^, Al^3+^, and [CaP_3_O_10_]^3−^ ions in the cement slurry form an electric double-layer structure on the surface of the cement particles. According to the electric double-layer theory, an electric double layer of adsorption and diffusion layers is formed when the particles have an electric charge on the surface. Additionally, the magnitude of ζ-potential often depends on the thickness of the diffusion layer, i.e., the thicker the diffusion layer the higher the ζ-potential. The complex [CaP_3_O_10_]^3−^ has a very strong negative charge and forms the diffusion layer of the bilayer structure. When the amount of STPP added is little, the surface of cement particles shows the positive potential of the adsorbed layer [26]. As the addition of STPP increases, the thickness of the diffusion layer increases, and the surface of cement particles shows the negative potential of the diffusion layer. Therefore, the potential on the surface of cement particles changes from positive to negative after 5 min, 10 min, 15 min, and 20 min of stirring. As the STPP content increases from 0–0.2%, more and more [CaP_3_O_10_]^3−^ ions are present in the diffusion layer, and the absolute value of ζ-potential increases significantly. When the STPP increased to 0.4%, the absolute value of ζ-potential was less than 0.2% after 5 min and 10 min of stirring. This indicates that the [CaP_3_O_10_]^3−^ ions in the diffusion layer decreased, and the thickness of the diffusion layer decreased. By comparing the changes of ζ-potential on the surface of cement particles after 5 min, 10 min, 15 min, and 20 min of stirring, it can be analysed that the longer stirring time leads to the partial [CaP_3_O_10_]^3−^ ion detachment from the bilayer structure, the diffusion layer thickness decreases, and the intensity of ζ-negative potential becomes smaller. The dispersibility of cement is related to the ζ-potential, which is based on the principle that the greater the intensity of the ζ-negative potential, the greater the electrostatic repulsion on the surface of cement particles, and the better the dispersibility of cement at that time. From Figure 4, it can be seen that when the additional amount of STPP is 0.2%; it can make the best dispersion of cement and also improve the quality and use performance of refractory castables very well.

### 3.3. Rheological Properties

**Figure 5:**
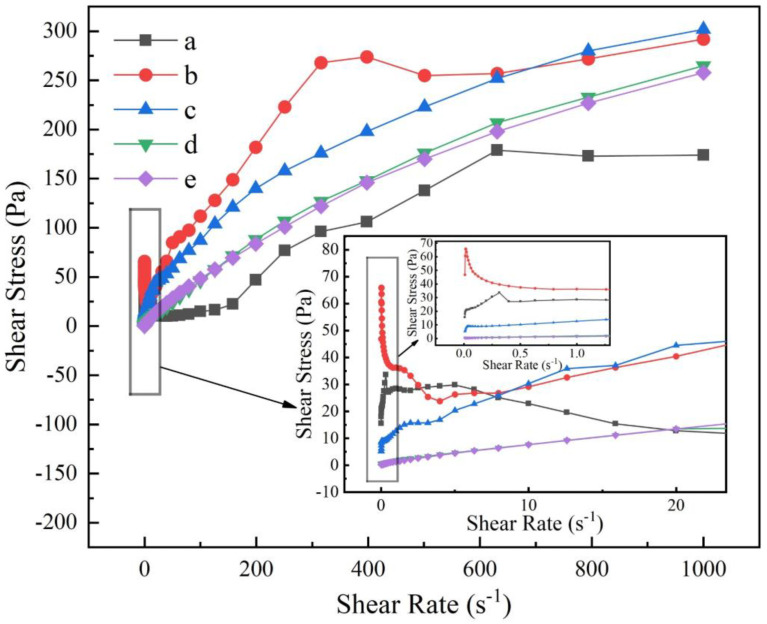
Shear stress versus shear rate curves for cement−water slurries added with different amounts of STPP: (a) without STPP, (b) with 0.05% STPP, (c) with 0.1% STPP, (d) with 0.2% STPP, and (e) with 0.4% STPP.

Figure 5 shows the variation of shear stress versus shear rate in the cement slurry with different amounts of STPP added. According to Figure 5, it can be seen that the shear rate basically does not change much when the shear stress changes abruptly without the addition of STPP and with the STPP addition of 0.05%. The former is due to the agglomerative water absorption effect and less dispersion between cement particles. The latter is due to the generation of very little [CaP_3_O_10_]^3−^, and the dispersion effect is not significant enough to overcome the yield stress. The order of yield stress is no STPP > 0.05% STPP > 0.1% STPP > 0.4% STPP > 0.2% STPP. When the shear rate is 1000 s^−1^, the shear stress gradually decreases as the content of added STPP increases. This law indicates that increasing the content of STPP can effectively prevent the agglomeration of cement particles and the generation of gel structure and improve the rheological properties of cement [27,28,29].

**Figure 6:**
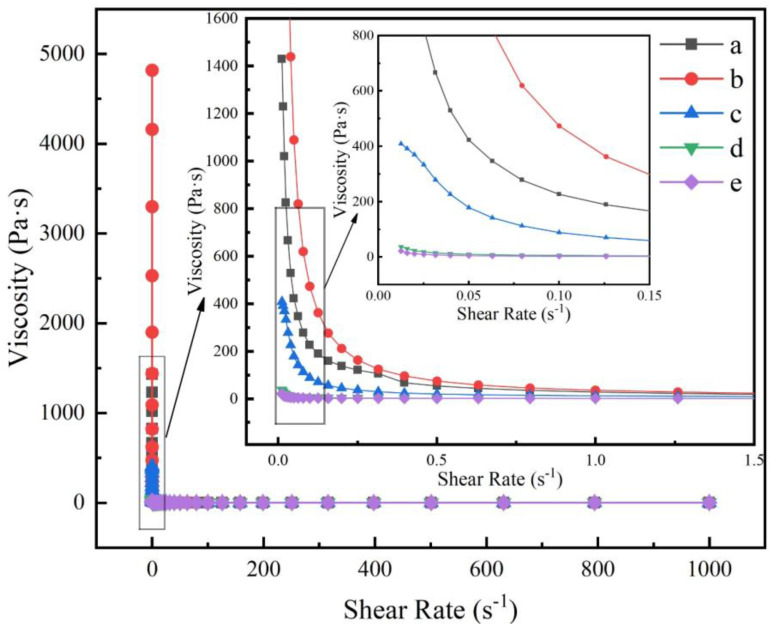
Viscosity−shear rate curves for cement−water slurries added with different amounts of STPP: (a) without STPP, (b) with 0.05% STPP, (c) with 0.1% STPP, (d) with 0.2% STPP, and (e) with 0.4% STPP.

Figure 6 shows the variation of the viscosity of the cement slurry with the shear rate for different amounts of STPP. As can be seen from Figure 6, when the shear rate is the same, the viscosity gradually decreases as the amount of STPP added increases. When the shear rate is between 0 and 0.5 s^−1^, the viscosity decreases rapidly with the increasing shear rate for no STPP added, 0.05% STPP added, and 0.1% STPP added, and the phenomenon of shear thinning occurs. At 0.2% and 0.4% of STPP, the viscosity hardly changes with the shear rate, indicating that this level of STPP can help to improve the rheological properties and serviceability of the PCAC cement slurries. A comprehensive analysis of Figure 5 and Figure 6 shows that the rheology of PCAC achieves optimum results and works best when STPP is added at 0.2%, thus enhancing the serviceability properties of the refractory castables.

### 3.4. Effect of SHMP Addition on Hydration Behaviour of Calcium Aluminate Cement

The hydration principle of pure calcium aluminate cements is that the initial components are chemically combined with water to produce hydration products, which then undergo a series of crystal transformations at different temperatures. Pure calcium aluminate cements contain CA and CA_2_, which produce the sub-stable hydration products C_2_AH_8_ and CAH_10_ at around 25 °C. At 25–35 °C, the sub-stable hydrates are rapidly transformed into the stable hydrates C_3_AH_6_ and AH_3_ [30,31,32].

**Figure 7:**
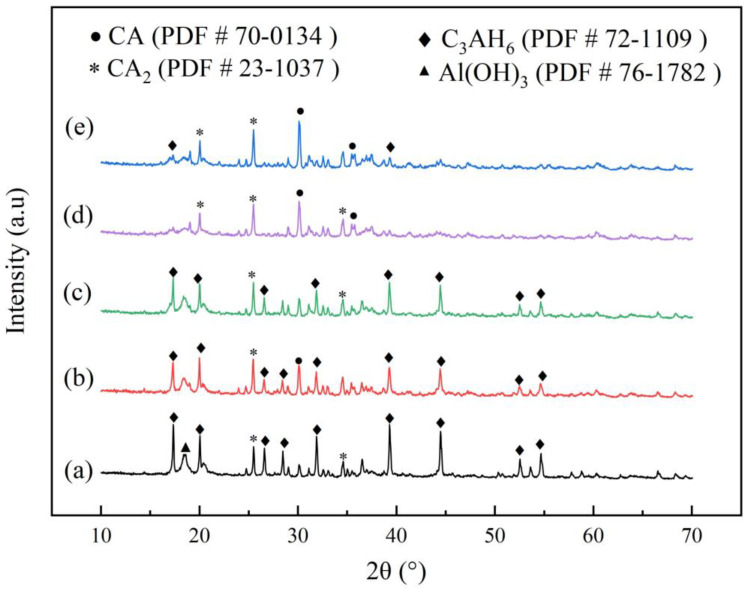
X−ray diffraction (XRD) patterns of PCAC after 1 d of hydration: (a) without STPP, (b) with 0.05% STPP, (c) with 0.1% STPP, (d) with 0.2% STPP, and (e) with 0.4% STPP.

**Figure 8:**
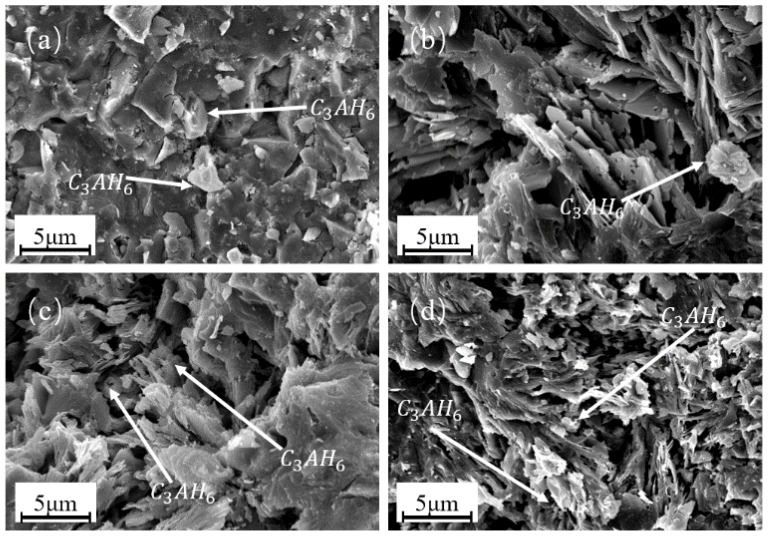
Scanning electron microscopy (SEM) images of PCAC after 1 d of hydration: (**a**) without STPP, (**b**) with 0.05% STPP, (**c**) with 0.1% STPP, and (**d**) with 0.2% STPP.

Figure 7 shows the X-ray diffraction (XRD) spectrum of PCAC after 1 d of hydration, which shows the effect of adding different amounts of STPP on the hydration process of PCAC. The XRD can only determine the type of substance in the sample, not the amount, but the relative content of the substance can be deduced from the peak area. As can be seen from the graph, the hydration products are mainly 3CaO·Al_2_O_3_·6H_2_O (C_3_AH_6_), with very small amounts of AH_3_, and the diffraction peaks of the samples without STPP are higher than those with STPP. By analysing the peak areas of C_3_AH_6_ (PDF # 72-1109), CA (PDF # 70-0134), and CA_2_ (PDF # 23-1037) in the diffractions, it was possible to deduce the hydration process of PCAC when different levels of STPP were added. When STPP was added at 0.05–0.2%, the content of C_3_AH_6_ gradually decreased, and CA and CA_2_ increased as the STPP content increased [33,34]. At a 0.2% STPP addition, little C_3_AH_6_ was produced. Figure 8 shows the SEM image of PCAC after 1 d of hydration. The morphology of C_3_AH_6_ is granular in the SEM image, and the trend of C_3_AH_6_ content can be judged by combining with the SEM image [35]. A combined analysis of the results in Figure 7 and Figure 8 shows that the addition of STPP inhibited the hydration of CA and CA_2_, especially at 0.2% of STPP, and there are the following explanations for this occurrence.

The STPP added to the cement slurry complexes with Ca^2+^ to form [CaP_3_O_10_]^3−^. The [CaP_3_O_10_]^3−^ adsorbs onto the surface of the cement particles, reducing the contact area between the cement particles and the water and inhibiting the hydration of CA and CA_2_. At 0.05–0.2% of STPP, the complexation reaction was promoted, and the amount of [CaP_3_O_10_]^3−^ adsorbed onto the cement particles gradually increased, and the inhibition of CA and CA_2_ hydration deepened. A total 0.2% of STPP resulted in the highest amount of [CaP_3_O_10_]^3−^ adsorbed onto the cement particles and the deepest inhibition of CA and CA_2_ hydration. At a 0.2–0.4% addition of STPP, the complexation reaction is inhibited, and less soluble complexes [CaP_3_O_10_]^3−^ are produced. Because the adsorption rate is lower at this point, fewer complexes are adsorbed on the surface of the cement particles, and the hydration of CA and CA_2_ is promoted. The results of the above analysis suggest that when selecting STPP as a dispersant for PCAC, the amount of STPP to be added should be controlled within a suitable range; otherwise, the hydration process of PCAC will be delayed, making the time for PCAC to set longer and affecting the performance of PCAC.

## 4. Conclusions

(1) STPP reacts with Ca^2+^ to form the complex [CaP_3_O_10_]^3−^. The [CaP_3_O_10_]^3−^ forms an electric double-layer structure with Ca^2+^ and Al^3+^ on the surface of the cement particles, which changes the potential of its surface and improves the rheology and dispersibility of cement through electrostatic repulsion. The increase in [CaP_3_O_10_]^3−^ adsorbed on the surface of cement particles reduces the contact area between cement particles and water, inhibits the hydration of CA and CA_2_, hinders the setting of cement particles, and delays the hydration process of cement.

(2) When STPP was added at 0.05%, the adsorption rate was 19%, and when STPP was added at 0.4%, the adsorption rate was 0%. When the additional amount of STPP was 0–0.2%, it promoted the complexation reaction, inhibited the dissolution of Ca^2+^, and reduced the Ca^2+^ content in the solution. When STPP was added at 0.2–0.4%, the complexation reaction was inhibited. The change in Ca^2+^ concentration in aqueous solution decreases first and then increases.

(3) Combined with the experimental results, it can be seen that the best combined performance on PCAC was achieved in summer (room temperature of 37 °C) when STPP was added at 0.2%.

(4) This study is a practical guide to the selection of the type of water-reducing agent for refractory castables and the amount to be added at different temperatures.

## Figures and Tables

**Table 1:** Chemical composition of PCAC.

	Al_2_O_3_	CaO	SiO_2_	Fe_2_O_3_	MgO	TiO_2_	SO_3_	Others
w/%	68.7	28.5	0.4	0.2	0.25	0.2	0.15	1.6

## Data Availability

Not applicable.
